# Standardizing Fetal Movement Monitoring using Count the Kicks

**DOI:** 10.1097/NMC.0000000000001048

**Published:** 2024-10-15

**Authors:** Adriane Burgess, Megan Aucutt, Sarah L. Coleman

**Affiliations:** **Adriane Burgess** is the Director of Innovation in Patient Safety and Quality at Maryland Patient Safety Center, Elkridge, MD. Dr. Burgess can be reached at aburgess@marylandpatientsafety.org; **Megan Aucutt** is the Program Director at Healthy Birth Day, Inc. The author can be reached at aucutt.megan@healthybirthday.org; **Sarah L. Coleman** is the State Expansion Director at Healthy Birth Day, Inc. The author can be reached at coleman.sarah@healthybirthday.org

**Keywords:** Fetal death, Fetal movement, Mobile applications, Prenatal education, Stillbirth

## Abstract

Stillbirth affects 1 in 175 pregnancies in the United States. There are significant racial and ethnic disparities in rates of stillbirth. Rates of stillbirth are highest among non-Hispanic Native Hawaiian or Other Pacific Islander and non-Hispanic Black women, more than twice the rate of non-Hispanic White women. Stillbirth is a public health crisis that warrants attention as it has significant physical, psychosocial, and economic effects on women and their family. Many stillbirths occur due to placental insufficiency, causing a lack of oxygenation of the fetus, which can result in decreased movement. Pregnant patients who experience stillbirth often observe decreased fetal movement days before birth. Daily fetal movement monitoring has the potential to identify pregnancies at risk so providers can intervene. Count the Kicks is a fetal movement monitoring program that provides standardized education and resources for expectant parents. Increased awareness of providers and childbearing families about the importance of fetal movement monitoring, standardized provision of education on fetal movement counting, and what to do if a baby's normal movement patterns change can be helpful in promoting healthy pregnancy outcomes.

## Abstract 2

Fetal movement counting is a patient-centered, low-risk, and low-cost intervention that has the potential to improve outcomes. There is little standardization in the education given to patients by clinicians on how to monitor fetal movements. There is no consensus among experts on a specific fetal movement counting method that works best. Patients need accurate easy to understand information on when to seek care. Count the Kicks provides resources and standardized education on fetal movement counting, promoting shared decision-making and patient empowerment.

**Figure:**
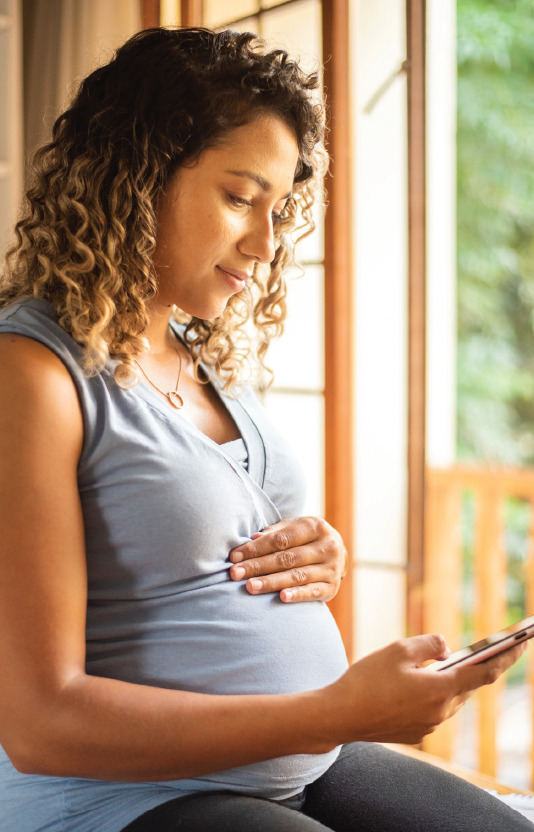
No caption available.

Stillbirth is defined as the death of a baby at or after 20 weeks gestation at any time before or during birth (American College of Obstetricians and Gynecologists [ACOG], 2020; Centers for Disease Control and Prevention [CDC], 2022a). Stillbirth affects about 1 in 175 births (CDC, 2022a). In 2021, there were approximately 21,000 stillbirths in the United States (CDC, 2022b). Since the 1940s, there has been a significant decline in stillbirth due to improvements in maternity care, yet since the early 2000s the rate has plateaued. (CDC, 2022b). There are significant racial and ethnic disparities in rates of stillbirth. Rates of stillbirth are highest among non-Hispanic Native Hawaiian or Other Pacific Islander and non-Hispanic Black women (CDC, 2022b). These rates are more than twice the rate of non-Hispanic White women (CDC, 2022b). Disparities in rates of stillbirth are perpetuated by factors such as chronic stress caused by racism in society and the health care system, social determinants of health, and lack of access to quality health care, all of which increase the risk of pregnancy complications, potentiate disparities, and increase risk of stillbirth (March of Dimes, 2020). There are several known risk factors for stillbirth (Table 1), yet most stillbirths in the United States occur in women with no risk factors (National Institute of Child Health and Human Development, 2023).

**TABLE 1.** RISK FACTORS FOR STILLBIRTH

**Sociodemographic Factors**	Maternal younger or older age Black maternal race (∗race is not a biological risk factor for stillbirth) Unmarried status Stress Low socioeconomic status
**Comorbid Conditions**	Chronic hypertension Pre-existing diabetes Systemic lupus Renal disease Antiphospholipid syndrome Obesity
**Obstetrical Conditions**	Nulliparity Multiple gestation History of stillbirth Oligohydramnios Cholestasis of pregnancy Preeclampsia Late-term or post-term pregnancy
**Fetal Conditions**	Intrauterine growth restriction Male fetal sex
**Other**	Pregnancy by assisted reproductive technology Smoking (including secondhand smoke) Substance use Environmental exposures (pollution or high temperature)

*Sources:* American College of Obstetricians and Gynecologists (2020) and National Institutes of Health (2023a, 2023b).

∗ Racism (rather than race) as experienced by many members of minoritized groups during their interactions with the health care system is the risk factor predisposing pregnant Black women to an increased risk of stillbirth.

Although it is unknown what causes all stillbirths, the leading causes of stillbirth include fetal genetic or birth defects, infection, complications with the umbilical cord or the placenta, and medical problems in the pregnant person such as hypertension or diabetes (National Institutes of Health, 2023a, 2023b; Nyarko et al., 2024). It is thought that many stillbirths occur due to placental insufficiency (ACOG, 2020). Due to the acute or chronic fetal hypoxemia associated with placental insufficiency, fetal movements may decrease as the fetus slows to conserve energy and a period of decreased movements may proceed fetal death (Bellussi et al., 2020; Mangesi et al., 2015; Pollock et al., 2020). Heazell et al. (2017) found that pregnancies that ended in stillbirth were more often associated with abnormalities in fetal movements in the preceding 2 weeks. Adequate fetal activity has been associated with overall fetal wellbeing (Pollock et al., 2020). Although decreased fetal movement is physiologically associated with fetal hypoxemia, evidence to date has not shown a reduction in stillbirth with the implementation of fetal movement monitoring (ACOG, 2021a, 2021b; Belluissi et al, 2020; Callaghan, 2018; Hayes et al., 2023; Norman et al., 2018). In a recent meta-analysis and systematic review of 16 randomized trials and 2 observational studies on encouraging maternal awareness of fetal activity and related management strategies, Hayes et al. (2023) concluded that randomized trials have not found a direct link between fetal movement awareness or counting and stillbirth, in part because stillbirth is a rare event, there is heterogeneity among interventions in published research, and many of the studies were underpowered to evaluate the main outcome, stillbirth. Though research has not shown that fetal movement monitoring should be routinely recommended to prevent stillbirth, increased awareness of and improved standardization of education on fetal movement monitoring may be of value (Callaghan, 2018). “Antenatal fetal surveillance is performed to reduce the risk of stillbirth” (ACOG, 2021b, p. e78).

## Fetal Movement Counting

Women pregnant with their first baby often first perceive fetal movements between 18 and 20 weeks gestation, whereas multiparous women perceive fetal movements between 16 and 18 weeks (Tsakiridis et al., 2022). Nulliparity, anterior placenta, increased body mass index, and advancing maternal age have been found to be associated with a delay in feeling fetal movement (Tsakiridis et al., 2022). Fetal movements are strongest between 28 and 34 weeks (Mangesi et al., 2015). Although fetal movement counting has been widely recommended to pregnant patients by nurses, midwives, and physicians during prenatal care since the 1970s and 80s, there is little standardization to whom and how fetal movement counting is taught (Barros et al., 2021; Bellussi et al., 2020; Daly et al., 2019; Hayes et al., 2023; Mangesi et al., 2015; Pollock et al., 2020). There are two main methods of fetal kick counting, the Sadovsky method and The Cardiff Count to Ten method. The Sadovsky method has the pregnant woman assess the number of movements that the baby has made over a period of time (i.e., 30 minutes or 2 hours), typically after meals (Mangesi et al., 2015). One of the most common fetal kick-counting instructions to patients is to call the provider if they feel less than 10 movements within 2 hours (Bellussi et al., 2020). The Cardiff method assesses the amount of time it takes to perceive 10 movements (Barros et al., 2021). Research has shown that adherence with fetal movement counting is better when the count-to-ten method is used (Barros et al., 2021). The differences in how patients are educated on fetal movement counting may affect their willingness and level of comfort to present for evaluation when fetal movement is decreased (Smyth et al., 2016).

There is also significant variation in how fetal movement counting has been studied. Researchers have used varying definitions of decreased fetal movement (ACOG, 2021b) as well as provided varying instructions on how and when patients should monitor fetal movement (Bellussi et al., 2020; Hayes et al., 2023). In some studies, pregnant women were given verbal instructions on fetal kick counting, and in others, they received written materials (Bellussi et al., 2020). In some studies, participants were told to monitor fetal movements daily beginning at 28 weeks, and in others, they were encouraged to count movements while in a certain position such as side-lying or to only count at a certain time of the day or after a meal (Bellussi et al., 2020). The variation in how fetal movement monitoring has been studied in research has made it challenging to synthesize the evidence on the impact of fetal movement counting on outcomes such as stillbirth (Hayes et al., 2023). Due to the lack of conclusive evidence, there is controversy about the widespread provision of education on fetal movement monitoring. In 2021, ACOG (2021a, 2021b) updated their recommendations for fetal kick counts as an antepartum fetal surveillance technique because of recent studies showing no benefit (Bellussi et al., 2020; Norman et al., 2018). A recent meta-analysis and systematic review had similar findings (Hayes et al., 2023).

Smith et al. (2021) report that between 4% and 23% of pregnant women present with reduced fetal movement during pregnancy. Maternal perception of a change in the strength of fetal movements may be clinically more important than a numeric decrease in fetal movements (Bradford & Maude, 2018). Instructions to patients on fetal movement counting often omit information on the importance of monitoring for changes in the strength of fetal movements. Bradford and Maude (2018) found that women often report increased strength of fetal movement at term. Encouraging patients to monitor trends in both the strength and frequency of fetal movements allows them to better characterize what is normal for their fetus (es) and seek care when there is a change (Bradford et al., 2019).

Fetal movement counting is a patient-centered, low-risk and low-cost intervention. Fetal movement counting has the potential to identify babies at risk of stillbirth by allowing them to seek immediate obstetric care so that the fetus can be evaluated and delivered if it is determined that the fetus is under stress (Huecker et al., 2023). As with any intervention aimed at improvement, a standardized approach to fetal movement counting is necessary to improve outcomes (Pollock et al., 2020). ACOG (2020) does not have specific recommendations on how to teach patients to count fetal movements; however, they suggest best practices include shared decision-making, encouraging patients to be aware of fetal movement patterns, being attentive to patients reporting reduced fetal movements being and using a systematic process to address their concerns. The Association of Women's Health, Obstetric and Neonatal Nurses (AWHONN) issued a practice brief on decreased fetal movement encouraging nurses to provide patient education about fetal movement, assess for fetal movement during every interaction with pregnant patients in their third trimester, and use shared decision-making (AWHONN, 2024).

Regardless of whether health care providers teach pregnant patients about fetal movement counting, many pregnant women informally monitor their fetus' movement (Smith et al., 2021). When determining if they should seek care for decreased fetal movement women often seek guidance from friends and relatives; therefore, education on fetal movement counting is important for everyone (Andrén et al., 2023). Family, friends, and health care providers may provide inaccurate information on fetal movement and fetal movement counting (Andrén et al., 2023). This can affect how pregnant women interpret and act on changes in their fetus' movements (Andrén et al., 2023). Standardized patient education on what to expect, how to monitor, and when to report changes in fetal movements is necessary. Count the Kicks is an evidence-based program that provides standardized fetal movement monitoring resources and education.

## Count the Kicks

Healthy Birth Day, Inc., is a 501(c)(3) organization dedicated to the prevention of stillbirth. In 2007, the founders of the organization learned of an observational public health study in Norway that demonstrated a 30% reduction in stillbirth by teaching pregnant women how to monitor fetal movement during the third trimester of pregnancy by tracking fetal movement counts daily (Tveit et al., 2009). Based on that research, the Count the Kicks program was created and implemented for the first time in Iowa in 2008. Count the Kicks provides tools, resources, and education to empower expectant families to speak up if they notice changes in their baby's movement. To implement Count the Kicks in Iowa, the leaders of Healthy Birth Day, Inc. worked closely with the Iowa Department of Health and Human Services to educate providers, expectant parents, and families throughout the state about the importance of fetal movement counting in the third trimester. Working together, they raised awareness about the program using public service announcements and social media, and by mailing educational materials directly to all birthing centers and maternity clinics throughout the state. Educational sessions on the Count the Kicks program were held for maternity care clinicians in health systems, community-based, and home health organizations. A web-based version of the app was first created in 2013. Beginning in 2015 when the mobile app was first developed through May of 2024, over 600,000 people have downloaded the app and almost 400,000 have registered, meaning they have input their contact information and set up a profile in the app. Individuals have registered in all 50 states. Since 2012, over 4 million pieces of educational materials have been mailed out across the United States.

### Fetal Movement Counting using Count the Kicks

The Count the Kicks program encourages women to begin monitoring their baby's movements starting in the third trimester at approximately 28 weeks, and at approximately 26 weeks for those designated as high-risk, such as women with multiple gestations. The program provides several ways to track fetal movements including a digital app, printable chart, web-based counter, and a bracelet that can be used to mark each movement during a counting session.

**FIGURE 1.**
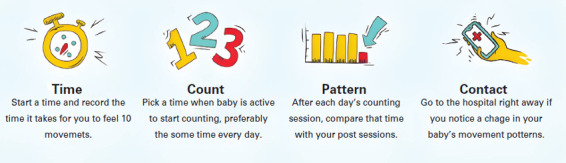
COUNT THE KICKS INSTRUCTIONS ON FETAL MOVEMENT TRACKING

**Instructions**. If not using the app, women are asked to start a timer and record the time it takes to feel 10 movements. Movements include rolls, kicks, jabs, swishes, pushes, anything except for hiccups as these are considered involuntary movements. There is no set time by which movements should be felt. A foundational principle of Count the Kicks is recognizing that every baby is different and the average amount of time it takes to achieve 10 movements varies. Counting fetal movements each day at around the same time allows patients to become familiar with what is normal activity for their baby and identify trends in their baby's movements. As part of the program, patients are educated to start counting when the baby is active. After each daily counting session, they are encouraged to compare the time it took to achieve 10 movements with past sessions and determine if there were any changes in fetal movements from previous sessions. Changes in fetal movements can include less movement, weaker movements, or an unusual or rapid increase in movement (Heazell et al., 2017). If a woman notices a change in their baby's movements, they should be educated to go to the hospital for evaluation (Figure 1) and it be reinforced that they can call or come in right away, even if after hours.

**FIGURE 2.**
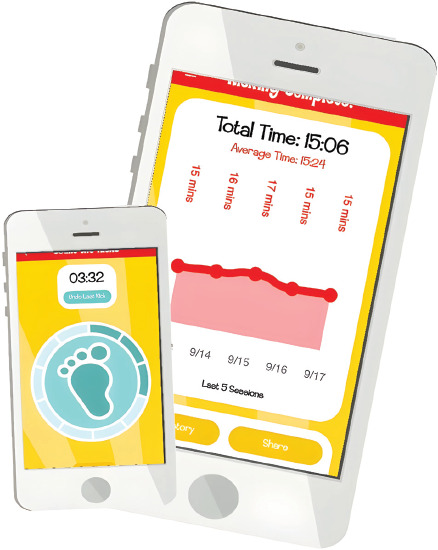
COUNT THE KICKS APP WITH KICK COUNTER AND GRAPH OF TIME TO ACHIEVE 10 MOVEMENTS TENDED OVER 5 DAYS

**The Digital App**. The *Count the Kicks mobile app* was developed in 2015 to optimize technology to assist pregnant women to track fetal movement over time. The app is free and is available for iPhone and Android and can also be downloaded on the Apple Watch. It is available in 21 languages. Singleton or twin pregnancies can be tracked using the app. Fetal movements are tracked by tapping the screen each time a fetal movement is felt. The app has an embedded timer that starts when the screen is tapped and marks each fetal movement. When 10 fetal movements are documented, the timer stops. The app includes a strength feature that encourages users to rate the strength of their baby's movements on a scale of 1 to 5, with 1 being “fluttery” and 5 being “fierce.” Once the fetal movement counting session is complete, the app user is shown a graphical outline of the number and timing of movements that occurred during their session (Figure 2). After two sessions, the app calculates and shows the average amount of time it takes the baby to achieve 10 movements and the average strength. This graph provides a visual representation of trends in baby's movements over time. Data on trends in fetal movement can be downloaded by the patient from the app and sent via text or email for the health care provider to review. The app includes automatic notifications sent at 24, 48, and 72 hours and 1 week after the last documented kick-counting session to remind users to monitor fetal movement. There is a notes section where users can document anything else they feel pertinent.

There are several additional features in the app such as a continue counting function, and a zip-code-based resource database to search for free and reduced-cost services, such as childcare assistance, housing assistance, and diaper banks among other services. In 12 states, the Count the Kicks app is configured to include a voluntary survey to assess social determinants of health such as access to transportation, food, or necessary medications. If gaps in access are noted, the user is provided contact information for local resources that address their area of need. Count the Kicks has ways for individuals to monitor fetal movement that do not require technology, such as paper kick counting charts and fetal movement monitoring bands.

### Dissemination and Implementation of the Program

There are opportunities for clinicians who provide prenatal education to use Count the Kicks materials to standardize how they teach about fetal movement counting. The app is free of charge as are many of the printed materials. Although some Count the Kicks materials can be obtained at no charge online or by downloading the app, funding is necessary to support widespread implementation or awareness campaigns across states or health systems. Funding provides access to a more comprehensive set of tools to assist in the dissemination of the program to health care providers, birthing people, and their families. Specifically, funding supports additional printed educational materials, access to translation services to ensure printed materials are translated to meet the unique needs of the community served, access to online webinars and education presented by Count the Kicks staff, access to digital media to increase awareness of fetal movement monitoring via social media, and more. At this time, Count the Kicks is being implemented in 31 states through funding from state public health departments, managed care organizations, health systems, not-for-profit organizations, and other philanthropic efforts.

Heazell et al. (2023) evaluated the implementation of Count the Kicks in Iowa and compared rates of stillbirth there with the rates in three neighboring states (Missouri, Illinois, and Minnesota) over time between 2005 and 2018. They found a decrease in the stillbirth rate in Iowa but not the other three states. However, they were unable to causally link a decrease in stillbirths to use of the app, education, resources on kick-counting, or raised awareness of stillbirth prevention and noted that most of the decline in stillbirths occurred before the app was available (Heazell et al., 2023). They reported app use in Iowa ranging from .081% in 2013 to 6.5% in 2018 based on app users and number of births and attributed the increase in app use to the media campaign on fetal movement awareness (Heazell et al., 2023). There were app users in the other three states but in much smaller numbers (Heazell et al., 2023). App users most likely represented only a fraction of women who were exposed to information on fetal movement counting as Iowa's statewide implementation of Count the Kicks included multiple outreach methods (Heazell et al., 2023).

**Site-Level Implementation**. Although implementation plans vary by site, Healthy Birth Day, Inc. recommends creating standard workflows to ensure materials reach pregnant women and that they receive consistent messaging on the importance of fetal movement counting and how to track movements. After ordering educational and promotional materials, sites should designate a site champion to lead the work and create a plan to standardize implementation by encouraging all clinicians who work with expectant parents to complete education on how to teach expectant parents about the program and fetal movement monitoring. Formalizing workflows to ensure expectant patients receive education on how to track fetal movement and are asked about fetal movement at every interaction in the third trimester is recommended. Systems to evaluate implementation to ensure the standardized provision of fetal movement education to all patients using designated workflows should be implemented.

Sites implementing the Count the Kicks program should consider including instructions on fetal movement counting in the after-visit summary or discharge instructions in the electronic health record. Education on fetal movement counting should be included in childbirth education programs, integrated into all third-trimester prenatal visits, and provided during triage. Hospitals and offices should consider sharing Count the Kicks messaging in waiting rooms and on phone “hold” messaging and encouraging patients to download and use the app.

## Clinical Implications

Stillbirth represents a significant public health crisis, yet there is a notable absence of federally funded national campaigns dedicated to its prevention. Seven times the number of babies lost to sudden unexpected infant death are lost to stillbirth each year (CDC, 2023a, 2023b). Prevention efforts are hindered by the lack of research on preventative strategies that are effective in reducing incidence of stillbirth. Public health initiatives have demonstrated remarkable success in enhancing outcomes, exemplified by the CDC Safe to Sleep campaign, which has substantially reduced sudden infant death syndrome fatalities over time (Wright, 2017). There are public health campaigns aimed at reducing rates of stillbirth in the Netherlands and Australia. Chan et al. (2023) and Chan et al. (2021) evaluated Australian public health campaigns aimed at stillbirth prevention and found that after implementation, clinicians were almost three times more likely to recommend patients with decreased movement come in for evaluation and women had increased awareness of modifiable behaviors aimed at preventing stillbirth including quitting smoking, being aware of baby's movements and going to sleep on the side. Page et al. (2018) estimated approximately 22% of stillbirths were deemed to be preventable based on their analysis of 512 stillbirths from 2006 to 2008 enrolled in the Stillbirth Collaborative Research Network. They concluded causes of potentially preventable stillbirth included placental insufficiency (12.7%), medical complications of pregnancy (6.1%), hypertensive disorders of pregnancy (3.9%), preterm labor (3.1%), intrapartum care (1.8%), and multiple gestations (0.8%; Page et al. 2018).

**Anxiety**. Some clinicians believe awareness of fetal movements should not be encouraged (Gidlöf, 2019). Reasons commonly cited include concerns that fetal movement counting increases patient anxiety (AlAmri & Smith, 2021; Delaram & Shams, 2016), may result in unnecessary intervention, and that it has not been shown to improve maternal and fetal outcomes (Bellussi et al., 2020; Hayes et al., 2023). Studies have found that fetal movement counting does not negatively affect maternal psychological or emotional status but rather improves maternal–fetal attachment (AlAmri & Smith, 2021). Delaram and Shams (2016) found that those who performed fetal movement counting from 28 to 37 weeks gestation had significantly lower state and trait anxiety scores than those who did not. In a study of those who used the Count the Kicks app, 77% stated the app helped lower their anxiety and 84% stated the app helped them bond with their baby (Buckingham-Schutt et al., 2022).

**Outcomes and Unnecessary Intervention**. The evidence has not shown improvements in the reduction in stillbirth through fetal movement counting (ACOG, 2021a, 2021b; Bellussi et al., 2020; Norman et al., 2018). However, a recent meta-analysis by Hayes et al. (2023) analyzed data from 18 studies and found that while encouraging awareness of fetal movement was not associated with a decrease in stillbirths, it may be associated with a reduction in NICU admission and Apgar scores <7 at 5 minutes, without an increase in rates of cesarean birth or induction.

**Decreased Fetal Movement as an Urgent Maternal Warning Sign**. The Alliance for Innovation on Maternal Health (2020) and the CDC (2022c) Hear Her campaign list slowing or stopping of a baby's movements as an urgent maternal warning sign. If a patient notices that their baby has stopped moving or is moving less than before, they should seek medical care immediately (CDC, 2022c). They explain “there is no specific number of movements that are considered normal, a change in your baby's movement is what is important” (CDC, 2022c, p. 1). Smith et al. (2021) reported that pregnant women appreciated the opportunity to learn about fetal movement counting from their maternity care givers and preferred receiving printed educational materials on the topic so they could refer to them later if needed.

ACOG (2021b) states that “for a pregnant individual reporting decreased fetal movement after viability, one-time antenatal fetal surveillance at the time the decreased movement is reported may be considered” (p. e183). Hospitals should have protocols that outline how to clinically evaluate pregnant women who present with decreased fetal movement (Barros et al., 2021) and have resources available to educate them on how to monitor fetal movement.

**Self-Advocacy and Patient Empowerment**. Count the Kicks encourages all expectant parents to listen to their bodies and not delay in contacting their provider if they feel there has been a change in their baby's movements. Research has shown that women feel their concerns about changes in their fetus' movement will not be taken seriously by their health care team (Smith et al., 2021). The program empowers patients by providing them with comprehensive education on fetal movement monitoring and the tools to do so therefore supporting self-advocacy and shared decision-making (Bradford et al., 2023). Data on the baby's movements can serve as a helpful tool, especially during telehealth appointments, and be used to help clinicians make decisions about the need for antenatal testing when there has been a change in the baby's movement.

Although stillbirth can result from underlying health conditions, it can also significantly affect maternal outcomes and increase the risk of severe maternal morbidity (Nyarko et al., 2024). Nurses should view stillbirth as a risk factor for many maternal physical and psychological complications (Burden et al., 2016; Wall-Wieler et al., 2019). Maternity nurses should ensure that all pregnant women receive standardized education about fetal movement counting, knowing their baby's normal movement patterns, and contacting their provider if movement patterns change (AWHONN, 2024). This education can be given in obstetric triage and at discharge to all patients in their third trimester of pregnancy. Nurses who work in outpatient antenatal settings such as maternal–fetal medicine, obstetric offices, and community settings can discuss this topic at every visit in the third trimester. Recognizing the significant gap in research in this area and the impact of stillbirth on society, childbearing women, and their families, nurse scientists should consider how to design studies to safely assess the impact of standardized fetal movement education on patient empowerment, shared decision-making, and maternal and neonatal outcomes (Stillbirth Working Group, 2023).

### Acknowledgment

Dr. Burgess' employer received $5000 from Healthy Birth Day, Inc. to cover her time spent in the development of the manuscript with coauthors. Both Ms. Aucutt and Ms. Coleman work for Healthy Birth Day the not-for-profit which leads Count the Kicks.

## CLINICAL IMPLICATIONS

- The Association of Women's Health, Obstetric and Neonatal Nurses encourages nurses to educate all pregnant women about how to monitor fetal movements and to assess fetal movement at every interaction in the third trimester.
- Count the Kicks provides resources and education in a variety of languages to ensure the equitable provision of fetal movement education.
- Patients who experience stillbirth often notice a reduction in fetal movement in the 2 weeks preceding death. It is important to listen carefully to patients' reports of changes in fetal movement and ensure they are evaluated immediately.
- Slowing or cessation of fetal movements is an urgent maternal warning sign. Patients should be given tools to assist with identifying and communicating this urgent maternal warning sign.
- There is variation in how patients are educated on fetal movement monitoring which can affect improved outcomes. Nurses should work within their teams to standardize how education about fetal movement counting is provided.

## INSTRUCTIONS Standardizing Fetal Movement Monitoring using Count the Kicks

### TEST INSTRUCTIONS

- Read the article. The test for this nursing continuing professional development (NCPD) activity is to be taken online at www.nursingcenter.com/CE/MCN. Tests can no longer be mailed or faxed.
- You'll need to create an account (it's free!) and log in to access My Planner before taking online tests. Your planner will keep track of all your Lippincott Professional Development online NCPD activities for you.
- There's only one correct answer for each question. A passing score for this test is 8 correct answers. If you pass, you can print your certificate of earned contact hours and access the answer key. If you fail, you have the option of taking the test again at no additional cost.
- For questions, contact Lippincott Professional Development: 1-800-787-8985.
- Registration deadline is Sept. 4, 2026.

### PROVIDER ACCREDITATION

Lippincott Professional Development will award 2.5 contact hours for this nursing continuing professional development activity.

Lippincott Professional Development is accredited as a provider of nursing continuing professional development by the American Nurses Credentialing Center's Commission on Accreditation.

This activity is also provider approved by the California Board of Registered Nursing, Provider Number CEP 11749 for 2.5 contact hours. Lippincott Professional Development is also an approved provider of continuing nursing education by the District of Columbia, Georgia, West Virginia, South Carolina, New Mexico, and Florida, CE Broker #50-1223. Your certificate is valid in all states.

**Disclosure:** The authors and planners have disclosed no relevant financial relationships regarding this educational activity.

**Payment:** The registration fee for this test is $24.95.
